# Supplementary material for: Clinical and Imaging Features of COVID-19-Associated Pulmonary Aspergillosis
Source: Diagnostics (Basel). 2022 May 11;12(5):1201. doi: 10.3390/diagnostics12051201 (PMC9140144; doi:10.3390/diagnostics12051201)
Supplement: Supplementary file 1 [file diagnostics-12-01201-s001.zip › diagnostics-1694570-supplementary.pdf]

Supplementary Table S1 Results of the mixed models (involvement and main pattern) COVID-19 versus CAPA.

**Total Lung Involvement**	**Estimate [b]**	**95% CI**	***p*-Value**
Intercept [a]	55.45	47.81–63.09	<0.001
CAPA: yes	2.09	−14.13–18.30	0.80
Days after symptom onset [d]	1.06	0.60–1.53	<0.001
Days after symptom onset*CAPA [e]	−0.44	−1.25–0.38	0.29
**Consolidation**	**Odds ratio [c]**	**95% CI**	***p*-value**
Intercept	0.08	0.02–0.28	<0.001
CAPA: yes	5.05	0.86–29.55	0.07
Days after symptom onset [d]	1.08	1.02–1.14	0.01
Days after symptom onset*CAPA [e]	0.92	0.87–0.99	**0.02**
**Crazy paving**	**Odds ratio [c]**	**95% CI**	***p*-value**
Intercept	0.10	0.03–0.37	<0.001
CAPA: yes	2.86	0.49–16.58	0.24
Days after symptom onset [d]	1.07	1.00–1.13	0.05
Days after symptom onset*CAPA [e]	0.95	0.89–1.02	0.18
**Ground glass opacity**	**Odds ratio [c]**	**95% CI**	***p*-value**
Intercept	4.32	1.68–11.07	0.002
CAPA: yes	0.15	0.03–0.69	0.01
Days after symptom onset [d]	0.89	0.84–0.95	<0.001
Days after symptom onset*CAPA [e]	1.11	1.02–1.19	**0.006**

Results of lung involvement, consolidation, crazy paving and ground glass opacity as outcomes and pulmonary aspergillosis, time, and the interaction between pulmonary aspergillosis and time as fixed effects. [a] The intercept gives the estimated mean total lung involvement when all covariates are zero, i.e., the estimated mean total lung involvement for COVID-19 patients at zero days after symptom onset is 55.45%. [b] The estimate represents the regression coefficient that is interpreted similarly as in a linear regression, i.e., the estimate gives the mean change in the outcome for a one-unit increase in the covariate. [c] The odds ratio is interpreted similarly as in a logistic regression, i.e., the odds ratio gives the estimated change in the odds of showing a specific main pattern (consolidation, crazy paving, or ground glass opacity) for a one-unit increase in the covariate. [d] The time variable (Days after symptom onset) gives the estimate for the mean change in the outcome (e.g. lung involvement) for each one-day increase after symptom onset for COVID-19 without pulmonary aspergillosis. [e] Days after symptom onset*CAPA is the estimate of the interaction between time and group and has to be added to the other regression coefficients for CAPA and when days after symptom onset is not zero.

Supplementary Table S2 Results of the mixed models (involvement and main pattern) COVID-19 with and without bacterial superinfection and CAPA.

**Consolidation**	**Odds Ratio**	**95% CI**	***p*-Value**
Intercept	0.394	0.11–1.44	0.16
COVID19-bacterial infection: yes	0.342	0.05–2.28	0.27
COVID19-no bacterial infection: yes	0.036	0.002–0.54	**0.02**
Days after symptom onset	0.997	0.96–1.03	0.86
Days after symptom onset*COVID19-bacterial infection	1.043	0.97.1.22	0.25
Days after symptom onset*COVID19-no bacterial infection	1.234	1.06–1.43	**0.006**

Results of consolidation as outcome pulmonary aspergillosis, time, and the interaction between pulmonary aspergillosis and time as fixed effects. For explanations please see supplementary Table S1.
